# A data-driven machine learning approach for brain-computer interfaces targeting lower limb neuroprosthetics

**DOI:** 10.3389/fnhum.2022.949224

**Published:** 2022-07-19

**Authors:** Arnau Dillen, Elke Lathouwers, Aleksandar Miladinović, Uros Marusic, Fakhreddine Ghaffari, Olivier Romain, Romain Meeusen, Kevin De Pauw

**Affiliations:** ^1^Human Physiology and Sports Physiotherapy Research Group, Vrije Universiteit Brussel, Brussels, Belgium; ^2^Brussels Human Robotics Research Center, Vrije Universiteit Brussel, Brussels, Belgium; ^3^Équipes Traitement de l'Information et Systèmes, CY Cergy Paris University, Cergy, France; ^4^Institute for Kinesiology Research, Science and Research Centre Koper, Koper, Slovenia; ^5^Institute for Maternal and Child Health - IRCCS Burlo Garofolo, Trieste, Italy; ^6^Department Engineering and Architecture, University of Trieste, Trieste, Italy; ^7^Department of Health Sciences, Alma Mater Europaea - ECM, Maribor, Slovenia

**Keywords:** neuroprosthetics, brain-computer interface, machine learning, electroencephalography, data—driven learning, lower limb amputation

## Abstract

Prosthetic devices that replace a lost limb have become increasingly performant in recent years. Recent advances in both software and hardware allow for the decoding of electroencephalogram (EEG) signals to improve the control of active prostheses with brain-computer interfaces (BCI). Most BCI research is focused on the upper body. Although BCI research for the lower extremities has increased in recent years, there are still gaps in our knowledge of the neural patterns associated with lower limb movement. Therefore, the main objective of this study is to show the feasibility of decoding lower limb movements from EEG data recordings. The second aim is to investigate whether well-known neuroplastic adaptations in individuals with an amputation have an influence on decoding performance. To address this, we collected data from multiple individuals with lower limb amputation and a matched able-bodied control group. Using these data, we trained and evaluated common BCI methods that have already been proven effective for upper limb BCI. With an average test decoding accuracy of 84% for both groups, our results show that it is possible to discriminate different lower extremity movements using EEG data with good accuracy. There are no significant differences (*p* = 0.99) in the decoding performance of these movements between healthy subjects and subjects with lower extremity amputation. These results show the feasibility of using BCI for lower limb prosthesis control and indicate that decoding performance is not influenced by neuroplasticity-induced differences between the two groups.

## 1. Introduction

Lower limb amputation is a disruptive intervention that severely reduces a person's mobility, impairing daily activities such as walking (Kirkup, 2007). A possible way for people with an amputation to recover part of the lost mobility is with the use of a prosthesis that replaces the lost limb (van der Linde et al., 2004). A suitable prosthesis can mimic the behavior of the intact limb and feels natural to control to the user. However, current prostheses lack such natural-feeling control due to mostly using passive mechanisms or limited active control (Versluys et al., 2009).

With passive prostheses, the energy required to mimic the biomechanical properties of the limb is provided by springs and dampers (Johansson et al., 2005). The main advantages of such a passive design are their relative simplicity, low cost, and the sturdiness of the device. However, passive devices lack the ability to generate mechanical power without a prior movement to store the energy required for actuation. The amount of mechanical power generated can also not exceed the retrieved energy. Additionally, they cannot autonomously adapt to the user's needs in a changing environment or provide feedback on the current state of the device.

As an alternative, active prostheses attempt to solve these issues by having actuated joints that users can control consciously through a controller interface (Windrich et al., 2016). This requires the integration of several additional components such as a power supply to power the actuation system. In addition, computation hardware, which also consumes power, is required to run the device controller software. Finally, a suitable control strategy is necessary to interact with the control system. These additional components typically add substantial bulk and weight to the device. However, the benefits of using active prostheses, such as improved natural gait reproduction (Zhang et al., 2010) and reduced metabolic cost (Au et al., 2009; Martinez-Villalpando et al., 2011), generally outweigh the costs.

Control of active prostheses can be achieved with multiple modalities, possibly a combination of those. Common modalities include muscle activity measured with electromyography (EMG; Fleming et al., 2021), or non-biological modalities such as human joint position with inertial motion units and ground reaction forces through force sensors built into the device (Vu et al., 2020). The optimal choice of control modality depends on the design of the prosthetic device and the targeted user population. For example, EMG can only be measured from intact muscles, and both joint positions and ground reaction forces are detected after the onset of movement. Designing a suitable prosthetic control system requires to balance these factors (Tucker et al., 2015; Windrich et al., 2016; Vu et al., 2020).

With each of theses modalities, the delay caused by to the need for motion initiation is a major drawback. Another possible interaction modality that can avoid this delay is the brain-computer interface (BCI), also called brain-machine interface (Lebedev and Nicolelis, 2017). BCIs measure brain signals to decode the user's intent, providing an increased sense of agency that enables natural-feeling control (Caspar et al., 2021). BCI can detect intention from the moment that neural activity is generated, whereas other modalities can only detect user intention after the onset of user action. A commonly used brain signal for BCI is the electroencephalogram (EEG). EEG has high temporal resolution, relatively low cost, and can be measured with non-invasive sensors, making it the preferred choice for BCI (Ramadan and Vasilakos, 2017). However, using EEG for lower extremity prosthetic control is currently rare (Lennon et al., 2020), as decoding lower limb movement from brain signals is more challenging than upper body movements (Penfield and Boldrey, 1937; Gandhoke et al., 2019).

BCI methods for the lower extremities were previously used in the context of neurorehabilitation in stroke patients (Miladinović et al., 2020b) and patients with Parkinson's disease (Miladinović et al., 2020a). However, such studies are lacking in the context of lower limb amputation. To design effective and user-friendly BCI systems for lower limb prostheses, a comprehensive understanding of the neural mechanisms of lower limb movement is necessary. One of such mechanisms is the movement-related cortical potential (MRCP), which can be observed from the EEG signal when performing a movement (Shakeel et al., 2015; Olsen et al., 2021). Detection of these MRCPs can be used to decode the onset of lower-limb movements for BCI control (Liu et al., 2018; Marusic et al., 2022). However, the involved brain structures and resulting neural activity related to movement tend to adapt after amputation due to neuroplasticity (Molina-Rueda et al., 2019). Although this phenomenon is known, our current understanding of how these changes affect EEG activity, and therefore BCI decoding, is limited.

Alternatively, motor imagery (MI) can also be used to control a BCI system (Padfield et al., 2019). MI is generated when imagining performing a movement, but can also be observed in motor preparation when performing the actual movement (Jeannerod, 1994). Therefore, it is still relevant to include frequency bands in the EEG signal that are related to MI, even though the movements were executed and not imagined. MRCP activity occurs in the frequency band of 0–5 Hz (Shakeel et al., 2015), while MI activity is observed between 8 and 30 Hz (Pfurtscheller and Neuper, 2001). These frequencies fall within the delta (<4*Hz*), theta (4–7 Hz), mu (8–12 Hz), and beta (13–30 Hz) frequency bands, respectively (Niedermeyer and da Silva, 2005).

One of the main issues in this field of research is the distinct lack of available EEG data from both able-bodied individuals and individuals with a lower limb amputation who perform the same movements. Indeed, a recent review by Asanza et al. (2022) found that currently, only one paper investigates BCI control of lower limb movement in individuals with a lower limb amputation (Murphy et al., 2017), and even then, they only use BCI to lock/unlock the knee of the prosthesis. To the best of our knowledge, no open data are available for this setting. Furthermore, most studies on lower limb BCI focus on detecting the onset of movement or distinguishing movement of the left from the right limb (Asanza et al., 2022), which limits the possible interactions that are supported by the control system.

For this purpose, we chose to employ a data-driven approach to investigate the feasibility of lower limb BCI. Data-driven approaches focus on the data rather than the models used (Brunton and Kutz, 2019). This approach was found successful in the field of machine learning (ML) as a method to identify bias in models (Ntoutsi et al., 2020) and improve decoding performance with suitable data engineering (Zhang and Duraisamy, 2015; Olson et al., 2017; Brunton et al., 2020). In the context of EEG decoding, bias was shown to be an important issue due to changes in both mean and variance of the signal over different recording sessions of the same individual (Miladinović et al., 2021). This phenomenon causes the used EEG features to violate the assumption of independent and identically distributed data on which most ML methods rely (Bishop, 2006).

For this study, a data-driven analysis was performed by training different off-the-shelf ML pipelines that discriminate ankle dorsiflexion from knee extension. Detecting ankle extension and dorsiflexion could be relevant when driving a car. Knee extension (besides hip extension) is crucial when transitioning from a seated position to a standing position. These are movements that occur frequently in daily activities (Hyodo et al., 2017). Furthermore, we investigate possible neuroplasticity-induced differences in EEG decoding between individuals with an amputation and able-bodied individuals that are possibly relevant to designing a lower limb prosthetic BCI control system. Neuroplasticity resulting from lower limb amputation is a well-known phenomenon (Schwenkreis et al., 2003; Draganski et al., 2006; Jiang et al., 2015; Bramati et al., 2019). However, the effect of these changes on EEG data and subsequent movement decoding from this data are currently unknown.

By identifying the methods that provide optimal decoding performance and investigating differences between individuals with an amputation and able-bodied individuals, our objective is to identify possible approaches to including EEG as a control signal for lower limb prostheses. This should also highlight the requirements that must be fulfilled to build a successful BCI control system. Additionally, confirming the viability of methods that were successful for upper-limb decoding should facilitate the development of lower limb control systems, as components could be reused and the same principles could be applied.

## 2. Materials and methods

To investigate the effect of neuroplasticity on the decoding of lower limb movements from EEG, we gathered data from both participants with an amputation and able-bodied participants performing lower limb movements. Subsequently, we used the acquired data to train multiple BCI decoding pipelines in an attempt to identify the most promising approach to controlling a lower limb prosthesis with EEG.

### 2.1. Participants

Participants with unilateral transfemoral amputation (aged 25–75 years) were recruited and completed their rehabilitation. Medicare Functional Classification (Gailey et al., 2002) level K2-4 was required for this experiment. Adults with bilateral, transarticular knee or hip, or additional upper limb amputation were excluded, as well as participants with neurological disorders or with stump pains and wounds. A control group of able-bodied participants was recruited and matched with the intervention group on age, sex, and educational level. For each group, indicated by the first digit in the identifier, eight participants were recruited, totalling 16 participants (3 female, 13 male). Except for two participants (1 in each group), all included participants have a counterpart in the other group based on the previously mentioned matching criteria. This is indicated by the last digit in the participant identifier. All participants were written and verbally informed about the study protocol and gave their written consent. The study was carried out in accordance with the Declaration of Helsinki (World Medical Association, 2013) and was approved by the Medical Ethics Committee of the University Hospital of Vrije Universiteit Brussel (B.U.N. 1432020000041).

### 2.2. Data acquisition

At the beginning of each session the participant's head circumference was measured to select the correct EEG cap size followed by an anamnesis, biometrical measurements and the EuroQol-5D questionnaire measuring quality of life (Herdman et al., 2011). The participant remained seated for the whole session in a chair that was adjusted to ensure optimal comfort. After applying the EEG acquisition hardware, the room was darkened and the participant was instructed to keep their eyes closed to avoid external stimuli as much as possible. Before starting the experiment, a baseline EEG measurement was recorded for 3 min while the participant remained seated and kept their eyes closed.

**Figure 1:**
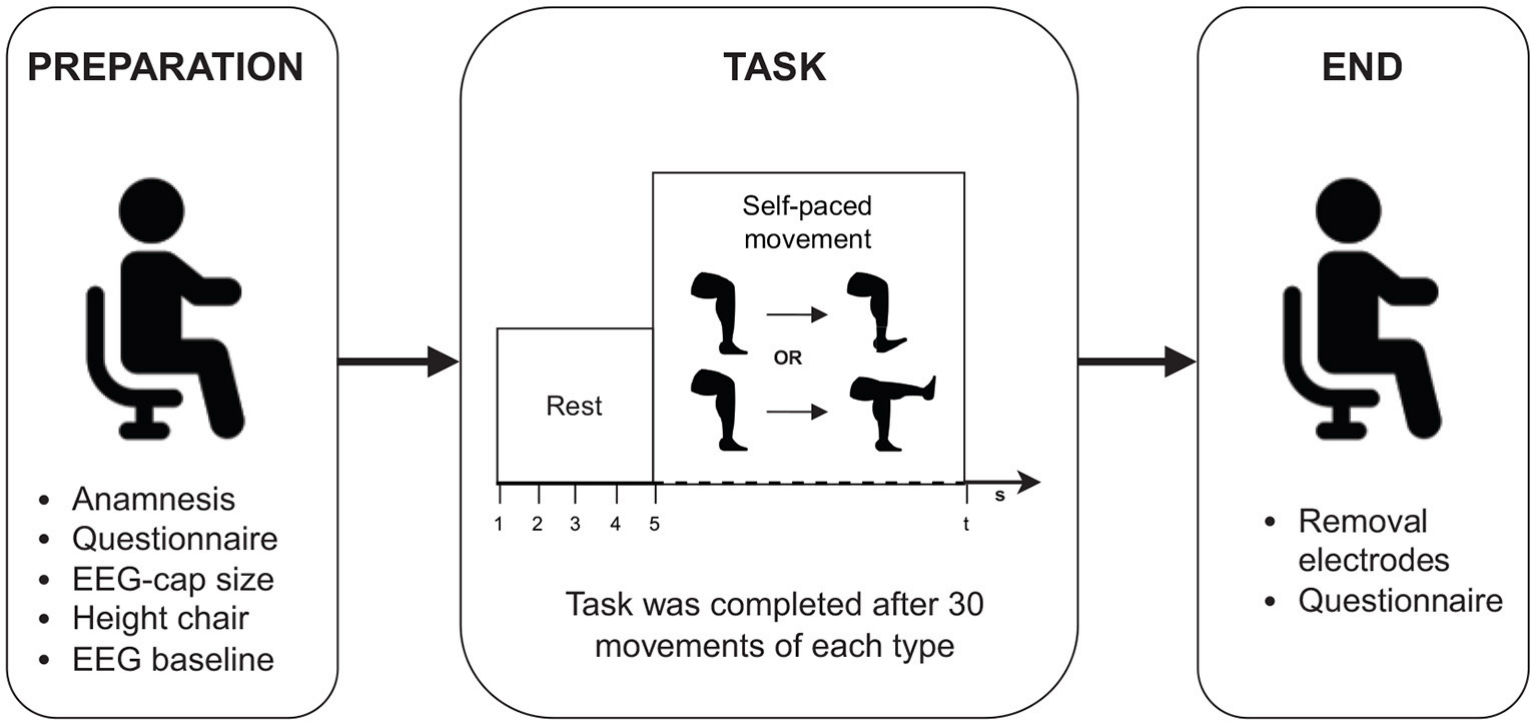
Overview of the experimental protocol.

Following preparation, the participant was instructed to perform multiple repetitions of a complete knee extension and ankle dorsiflexion at their own pace with their dominant leg. For participants with an amputation, this corresponded to their healthy leg. The participant was not informed about the number of expected repetitions that they had to perform to avoid that they would count their movements. They were informed to take approximately 5 s to rest between each movement and that the entire session should take approximately 15 min. The experimenter counted the number of movements performed until 30 repetitions were performed for each movement. When a participant reached the expected number of movement repetitions, they were told that the experiment was over. No additional feedback was provided. An overview of the experimental procedure is shown in Figure 1.

Markers were placed in the data during the experiment with a label that indicates the movement performed. After the experiment, the timing of each marker was updated based on auxiliary EMG data from sensors (Ambu^®^Neuroline 710, Ambu A/S, Denmark) that were placed on the rectus femoris and tibialis anterior. In addition to EMG, accelerometers were attached to the sternum, two bilateral sensors were installed on the anterior margo tibiae, and one sensor on the fifth metatarsal of the non-amputated foot. Both auxiliary signals were used to ensure precise timing of the movement-onset markers.

The acquisition of 64 channel EEG was performed using a LiveAmp amplifier (Brain Products GmbH, Germany) with actiCap snap active wet electrodes. The auxiliary signals were connected with the LiveAmp Sensor and Trigger Extensions to enable synchronization with the EEG signal. The EEG signals were recorded with 500 Hz sample frequency and were resampled to 128 Hz. Subsequently, the signal was preprocessed with a one-pass, zero-phase, non-causal bandpass filter with a finite impulse response designed using the windowed time-domain design (firwin) method. The window type is a Hamming window with a 0.0194 passband ripple and 53 dB stopband attenuation. The filter length was automatically determined for each filter on the basis of the reciprocal of the shortest transition bandwidth. Specifically, the reciprocal value (in samples) is multiplied by 3.3. Applied filter frequencies are presented in Section 2.4. Finally, the signal was epoched from −2 to 2 s relative to the movement onset, which is given by markers that were placed in the data, yielding labeled windows of 512 samples each.

### 2.3. Data processing

The feature extraction and classification models used in the decoding pipelines to discriminate ankle dorsiflexion from knee extension were chosen for their off-the-shelf availability and for their previous success in decoding upper extremity movements (Rashid et al., 2020). Although more advanced models exist, these methods are used in most of the literature (Hosseini et al., 2021) and are also relatively simple mathematically, enabling better explainability of the predictions that were made. More advanced models such as deep learning are currently outside of the scope of this study.

By combining different approaches and selecting optimal parameters for these models, we expect to find commonalities that are indicative of the optimal choice for decoding EEG from lower limb movements. This should highlight promising avenues of research for future work and enable the design of future general-purpose BCI systems.

To compare the EEG activity between the groups of individuals with an amputation and able-bodied individuals, epochs of both movements were averaged for each participant. For this analysis, the preprocessing of EEG was different to focus more on the frequencies in which MRCPs typically occur. The data was filtered to only keep frequencies between 0.1 and 5 Hz and the resampling was set to 250 Hz to allow for higher temporal resolution in this analysis. Additionally, bad epochs were rejected or repaired by using an automated method (Jas et al., 2016). Subsequently, the grand average was calculated over the individuals in each group. The resulting grand averages were then compared by aggregating sensor data and taking the average global field power (GFP; Lehmann and Skrandies, 1980; Murray et al., 2008) across each channel.

Data were processed with the Python 3 programming language (Van Rossum and Drake, 2009, RRID:SCR_008394), using the MNE toolkit to read and filter EEG data (Gramfort et al., 2013, RRID:SCR_005972). MNE was also used to epoch the data and extract features from the epoched EEG. Automated rejection and repairing of epoch data was handled by the AutoReject software library (Jas et al., 2017). The employed ML models were provided by the Scikit-learn software library (Pedregosa et al., 2011, RRID:SCR_002577), except for the neural network model, which was implemented using PyTorch (Paszke et al., 2019, RRID:SCR_018536). Custom code was written to perform the experiments and integrate the different software components of the software libraries mentioned above.

The hardware used to perform training and evaluation of the models was a desktop computer running on the Ubuntu 20.04 operating system. The machine has an AMD Ryzen 9 3900X 12-Core Processor and has 66 GB of memory. The neural network models were run on the machine's NVIDIA GV100 (TITAN V) graphical processing unit by leveraging the CUDA software toolkit (Luebke, 2008).

#### 2.3.1. Feature extraction

A commonly used feature in BCI is the Power Spectral Density (PSD) which is often used in neural signal analysis too (Bialek et al., 1996; Stoica and Moses, 2005; Bascil et al., 2016). This method projects the time-series signal into the time-frequency domain with a Fourier transform that is subsequently normalized. PSD indicates the amount of energy in each frequency band. For this experiment, PSD was computed with the multitaper method (Walden and Percival, 1993) using the default parameters provided by the MNE toolkit.

Another common feature extraction method is common spatial patterns (CSP) (Koles et al., 1990; Tortora et al., 2020a; Gao et al., 2021). This adaptive filtering method projects the original channel space onto a new lower-dimension space. The linear mapping is obtained by optimizing the variance (power) to be maximally informative with respect to two classes (knee extension vs. dorsiflexion in our case). The algorithm uses the preclass signal covariance matrices and solves the generalized eigenvalue problem to compute the patterns.

Finally, we also applied the xDAWN algorithm to feature extraction (Rivet et al., 2009, 2011). This algorithm is generally used to reduce the noise in the signal. This method is therefore more of a data cleaning method rather than feature extraction. The output is therefore the same shape as the input and typically needs further reducing, for which we use Principal Component Analysis (PCA).

#### 2.3.2. Machine learning classifiers

Logistic regression (Bishop, 2006) is a linear model of classification, and not regression as its name implies. Here, the probabilities of belonging to a class given the input are modeled using a logistic function. Training the model consists in finding the parameters of this function that minimize the classification error on the training data.

Linear Discriminant Analysis (LDA; McLachlan, 1992) is a classifier that consists of finding a linear combination of input features that enables the characterization or discrimination of two or more classes. LDA is an attractive classifier because it can be solved analytically, with a closed-form solution that is straightforward to compute. Additionally, it is inherently multiclass, has proven to work well in practice, and has no hyperparameters to tune.

Neighbor-based learning (Altman, 1992) is a form of instance-based learning or non-generalizing learning: it does not attempt to find optimal parameters for a general model but instead retrieves samples of the training data. Classification is determined by a majority vote of the nearest neighbors in the input space. In k-nearest neighbors classification, this decision is made by selecting the k (a user-provided parameter) nearest neighbors based on some distance measure, such as Euclidean distance for example. The optimal choice of the value k is highly data-dependent. Generally, a larger k suppresses the effects of noise, but makes the classification boundaries less distinct.

Support vector machines (SVM; Cortes and Vapnik, 1995) are a type of supervised learning model used for classification, regression, and outlier detection. SVM projects training inputs to points in a mathematical space so that if we draw a decision boundary between the points in this space, the gap between the groups is maximal. New inputs are then projected into that same space and assigned a label based on which side of the decision boundary they fall on. The decision boundary is linear, but SVMs can also use non-linear decision boundaries by using the kernel trick (Hofmann et al., 2008). SVMs have multiple advantages, including effectiveness in high dimensional spaces and in cases where the number of dimensions is greater than the number of samples. Other advantages are that it is memory efficient and versatile, as different kernel functions can be specified for the decision function. However, one of the main disadvantages is that SVMs do not directly provide probability estimates, requiring an expensive cross-validation step for this.

Random Forest (RF; Breiman, 2001) consists of an ensemble of decision trees where each tree in the ensemble is constructed from a subset of data drawn with replacement (i.e., a bootstrap sample) from the training data. Furthermore, either all input features or a random subset of fixed size, which is a hyperparameter of the model, are used to determine the split of each node in each decision tree. The purpose of these two sources of randomness is to decrease the variance of the forest estimator. This reduced variance sometimes comes at the cost of a slight increase in bias. In practice, the variance reduction is often significant, hence yielding an overall better model.

In extremely randomized trees (ExtraTrees; Geurts et al., 2006), randomness is further increased in the way the splits are calculated. Instead of looking for the most discriminative thresholds as in RF, thresholds are drawn at random for each candidate feature and the best of these randomly generated thresholds is employed. This usually enables a further reduction of the variance of the model, at the expense of a slightly greater increase in bias.

Neural networks (Rosenblatt, 1958) are a type of parametric model consisting of a network of interconnected neurons. Most neural networks only allow connections between neurons in a single direction and are called feedforward neural networks. When training a neural network, one tries to find the optimal set of weights that are associated to each connection, which are used to multiply the value from the outgoing neuron, yielding a new value as input for the incoming neuron. The most simple form of neural networks are called Multilayer Perceptrons (MLP; Rumelhart et al., 1985), also called vanilla neural networks, and consist of an input layer where each node receives one of the input values, one or more hidden layers where each neuron in a layer is connected to each neuron in the next layer, and finally an output layer that will contain the predicted value(s).

### 2.4. Decoding pipeline evaluation

The different combinations of feature extraction and ML methods used are listed in Table 1. For each of these combinations, a grid search was performed over the set of realistic parameter values for each component of the pipeline. Additionally, several filter boundaries were used for bandpass filtering in some of the pipelines. The included lower boundaries were 0.1, 5, and 8 Hz, and the candidate upper boundaries were 32, 35, and 41 Hz. The choices for these boundaries were based on common values found in literature, with the most common frequency band being 8–30 Hz (Singh et al., 2021; Asanza et al., 2022). These filter boundaries maintain the Mu and beta frequency bands, which are associated with motor activity (Pfurtscheller and Neuper, 2001). The lower boundaries of 0.1 and 5 HZ were chosen to investigate the effect of only filtering out low drift noise and keeping the MRCP-related theta band, respectively. Higher boundaries were empirically chosen to investigate the effect of including the gamma bands in a gradual way.

**Table 1:** Evaluated EEG decoding pipelines.

**Features**	**Classifier**	**Other steps**
PSD	RF	Vectorize and PCA between PSD and RF
PSD	KNN	Vectorize and PCA between PSD and KNN
PSD	LDA	Vectorize and PCA between PSD and LDA
PSD	Logistic regression	Vectorize and PCA between PSD and logistics regression
PSD	ExtraTrees	Vectorize and PCA between PSD and ExtraTrees
PSD	SVM	Vectorize and PCA between PSD and SVM
PSD	MLP	Vectorize, PCA and Datatype cast between PSD and MLP
CSP	RF	
CSP	KNN	
CSP	LDA	
CSP	Logistic regression	
CSP	ExtraTrees	
CSP	SVM	
CSP	MLP	Datatype cast before MLP
XDawn	LDA	Vectorize and MinMax Scale between XDawn and LDA
XDawn	Logistic regression	Vectorize and MinMax Scale between XDawn and LDA

*PSD, Power spectral density; CSP, Common spatial patterns; RF, Random forest; KNN, K-nearest neighbors; LDA, Linear discriminant analysis; ExtraTrees, Extremely randomized trees; SVM, Support vector machine; MLP, Multilayer perceptron; PCA, Principal component analysis*.

To evaluate model classification performance, a 10-fold cross-validation procedure was performed on the data from each participant separately (within-subject evaluation). The metrics used to evaluate the performance of the model are the average cross-validation accuracy and the average cross-validation F1 score. F1 score is a measure of binary classification accuracy which is calculated as the harmonic mean of precision and recall, where precision is the number of true positives divided by the number of samples that were classified as positive (Powers, 2020). Recall corresponds to the number of true positives divided by the number of samples that should be classified as positive.

The comparison of classification performance between the group of persons with amputation and the able-bodied group was made by performing an independent sample *t*-test that tests the null hypothesis that the average best pipeline decoding accuracy is the same for both groups.

## 3. Results

### 3.1. Characterization of the decoding pipelines

The best performing pipelines and their optimal parameter settings, based on accuracy, are shown in Tables 2, 3 for the group of individuals with an amputation and the group of able-bodied individuals, respectively. The mean results of the cross-validation and the mean standard deviation (SD) values of the cross-validation for each group are shown at the bottom of each table. In addition to accuracy scores, these tables also show F1 score results for the same pipeline and the optimal lower and upper bounds for the bandpass filter used for preprocessing.

**Table 2:** Decoding performance in individual participants with a lower limb amputation.

**Participant**	**Best pipeline**	**Best parameters**	**Best frequencies**	**Best accuracy**	**Best F1**
01	csp -> knn	csp__n_components: 4 knn__leaf_size: 5 knn__n_neighbors: 9 knn__p: 1 knn__weights: uniform	0.1–41 Hz	0.898 ± 0.033	0.901 ± 0.023
02	csp -> rf	csp__n_components: 8 rf__criterion: entropy rf__max_features: 0.75 rf__n_estimators: 40	8–35 Hz	0.744 ± 0.070	0.713 ± 0.049
03	psd -> vectorize -> pca -> extratrees	extratrees__criterion: entropy extratrees__max_features: 0.75 extratrees__n_estimators: 80 pca__n_components: nan pca__whiten: False	0.1–41 Hz	0.918 ± 0.075	0.902 ± 0.090
04	csp -> extratrees	csp__n_components: 6 extratrees__criterion: entropy extratrees__max_features: 0.5 extratrees__n_estimators: 160	5–32 Hz	0.850 ± 0.097	0.832 ± 0.117
05	csp -> knn	csp__n_components: 3 knn__leaf_size: 5 knn__n_neighbors: 7 knn__p: 1 knn__weights: uniform	8–32 Hz	0.774 ± 0.167	0.796 ± 0.143
06	csp -> rf	csp__n_components: 9 rf__criterion: entropy rf__max_features: 0.25 rf__n_estimators: 20	8–32 Hz	0.763 ± 0.118	0.679 ± 0.222
07	csp -> extratrees	csp__n_components: 8 extratrees__criterion: entropy extratrees__max_features: log2 extratrees__n_estimators: 20	8–32 Hz	0.954 ± 0.062	0.951 ± 0.066
09	csp -> rf	csp__n_components: 8 rf__criterion: gini rf__max_features: sqrt rf__n_estimators: 20	8–41 Hz	0.850 ± 0.082	0.849 ± 0.098
Mean				0.844 ± 0.088	0.828 ± 0.101
SD				0.072 ± 0.038	0.089 ± 0.058

**Table 3:** Decoding performance on individual able-bodied participants.

**Participant**	**Best pipeline**	**Best parameters**	**Best frequencies**	**Best accuracy**	**Best F1**
11	csp -> log_reg	csp__n_components: 5 log_reg__model__solver: liblinear	5–32 Hz	0.897 ± 0.108	0.897 ± 0.095
12	csp -> knn	csp__n_components: 3 knn__leaf_size: 5 knn__n_neighbors: 5 knn__p: 1 knn__weights: uniform	8–35 Hz	0.815 ± 0.104	0.797 ± 0.111
14	csp -> svm	csp__n_components: 7 svm__degree: 4 svm__gamma: scale svm__kernel: poly	8–32 Hz	0.947 ± 0.072	0.942 ± 0.079
15	csp -> extratrees	csp__n_components: 9 extratrees__criterion: gini extratrees__max_features: 0.75 extratrees__n_estimators: 160	5–32 Hz	0.763 ± 0.141	0.744 ± 0.186
16	csp -> extratrees	csp__n_components: 8 extratrees__criterion: entropy extratrees__max_features: sqrt extratrees__n_estimators: 40	5–35 Hz	0.892 ± 0.078	0.895 ± 0.066
17	psd -> vectorize -> pca -> extratrees	extratrees__criterion: entropy extratrees__max_features: 0.25 extratrees__n_estimators: 140 pca__n_components: nan pca__whiten: True	5–41 Hz	0.738 ± 0.078	0.756 ± 0.038
18	psd -> vectorize -> pca -> extratrees	extratrees__criterion: entropy extratrees__max_features: sqrt extratrees__n_estimators: 40 pca__n_components: nan pca__whiten: True	8–41 Hz	0.787 ± 0.050	0.768 ± 0.061
19	csp -> rf	csp__n_components: 3 rf__criterion: gini rf__max_features: 0.25 rf__n_estimators: 60	5–32 Hz	0.923 ± 0.049	0.920 ± 0.046
Mean				0.845 ± 0.085	0.840 ± 0.085
SD				0.074 ± 0.029	0.076 ± 0.044

From these tables we can observe that both groups reach an average classification accuracy of 84% and F1 scores were 0.828 and 0.840 for the group of individuals with an amputation and able-bodied individuals, respectively. The best performance was achieved for participant 07 with 94% accuracy and the worst performance was for participant 17, reaching 74% accuracy. There is no single best pipeline or frequency band that is the optimal choice for all participants. We also see that there does not seem to be any trend regarding optimal choices for either participant group.

**Figure 2:**
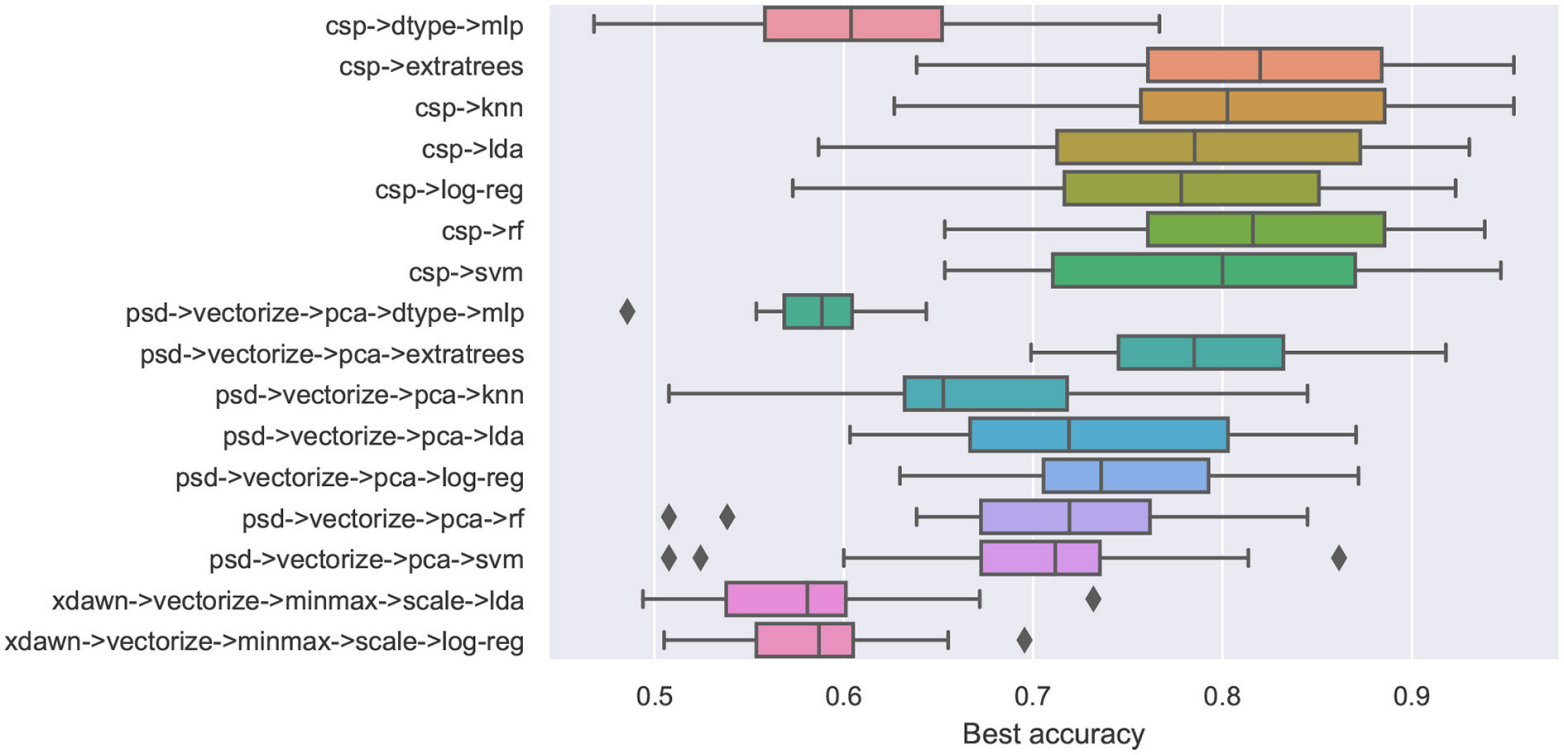
Average decoding accuracy on the cross-validation test set across all participants for every pipeline.

To characterize the consistency of decoding performance over all users, Figure 2 shows box plots for each considered pipeline based on the average test decoding accuracy over all participants.

From this figure, we can observe that the pipelines using CSP consistently achieve higher median accuracy compared to those employing other feature extraction methods. However, the pipelines that use PSD for feature extraction have lower variance, with the exception of the one that uses KNN for classification and outliers in both the ones that use RF and SVM for classification. Regarding classification methods, RF and its variant ExtraTrees achieve the highest median accuracy overall.

### 3.2. Differences between able-bodied participants and participants with an amputation

Regarding classification accuracy, there are no statistically significant differences between the amputee and able-bodied groups (*p* = 0.99). However, best average performers are different between the two groups, but no clear pattern emerges with regards to the best performing pipelines for the two groups.

**Figure 3:**
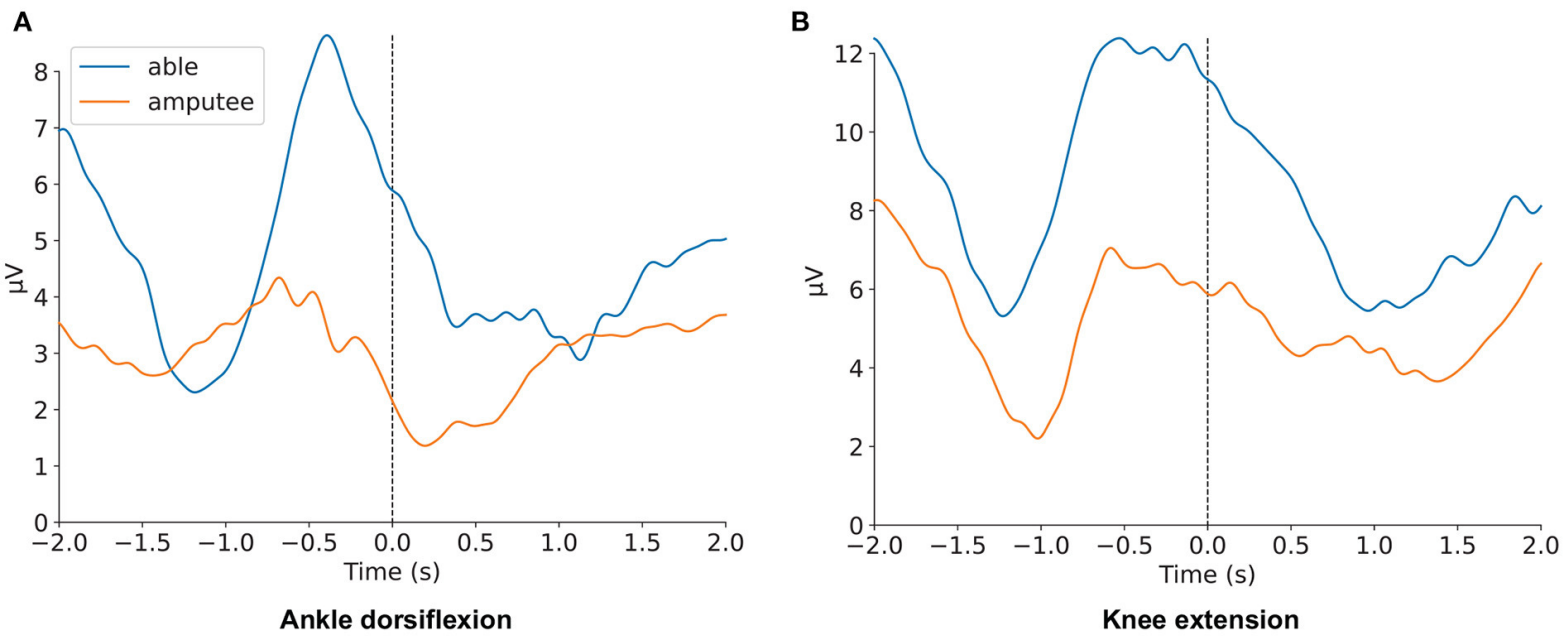
Comparison of grand average of the GFP activity between individuals with an amputation and able-bodied individuals when performing **(A)** ankle dorsiflexion and **(B)** knee extension.

Differences between GFP in able-bodied individuals and individuals with an amputation are shown in Figure 3 for ankle dorsiflexion and knee extension, respectively. The X-axis shows time with relation to the movement onset, which is indicated by the vertical dotted line at 0.0 s.

Figure 3 clearly displays the expected patterns related to motor preparation for both individuals with an amputation and able-bodied individuals during both movements. For ankle dorsiflexion, the GFP pattern in individuals with an amputation shows a deviation from able-bodied individuals.

## 4. Discussion

The principal aim of this study was to identify which methods are most suited to decode lower limb movement from EEG and to assess the general feasibility of using EEG as a control signal in a BCI system. Additionally, we investigated the differences between decoding movements from individuals with an amputation and able-bodied individuals to examine whether neuroplasticity induced by the lack of afferent and efferent neural processing in motor cortices in individuals with an amputation might result in different outcomes compared to healthy individuals. For this purpose, we compared the single-joint movement (knee extension vs. ankle dorsiflexion) decoding performance in both groups for a set of well-known decoding pipelines with off-the-shelf components. Single-joint movements were chosen for their importance in scenarios that deviate from regular gait, such as ascending stairs, and for their utility in daily activities such as driving a car or transitioning from a seated position to a standing position. The employed decoding methods were chosen for their simplicity and previous success in upper limb decoding. As the purpose of this study was to show the feasibility of lower limb movement decoding rather than to benchmark state-of-the-art methods, evaluation was restricted and comparison to decoding results in previous work was limited.

The results that are presented in Tables 2, 3 show that the resulting average individual classification accuracy was 84% for both groups and the average F1 score was 0.828 and 0.840 for the group of individuals with an amputation and the able-bodied group, respectively. With some accuracies reaching 94%, these results match the best performing methods in the literature, which often use more advanced algorithms and simpler classification tasks (Asanza et al., 2022). Variability is still an issue, and the lowest accuracy of 74% would be insufficient for clinical applications, but it is still acceptable in a BCI setting, taking into account that this could still be improved and that there are methods to mitigate the low single-trial classification performance (Rashid et al., 2020). While Murphy et al. (2017) showed the feasibility of BCI control for lower limb prostheses through a case-study, our data-driven study shows that accurate decoding of movements from EEG is possible given the right user-specific decoding pipeline.

Compared to decoding performance obtained in the literature that is reviewed in Asanza et al. (2022), which mostly use similar methods, the performance obtained in this article is in the same range. If we compare with the methods that decode MI presented in Gu et al. (2021), we can also see that our decoding results are on par and that using more advanced methods could yield further performance improvements. However, it is important to note that accuracy results cannot be directly compared with models that are trained on different datasets and potentially use a different training regime.

The results show that there is no single best pipeline to decode the movement of the lower extremities from EEG. This is in line with previous literature showing that interindividual variability makes it difficult to create a general-purpose BCI classifier (Lotte et al., 2018). For feature extraction, the best choice seems clear, as CSP is the feature extraction method in 13 of the 16 best pipelines. We have also observed that when using CSP as the feature extraction method, the median accuracy over all participants is higher. Moreover, XDawn never appears to be the optimal choice for features/feature extraction.

For classification, random forest methods, RF and ExtraTrees, appear to be the best classifier choice in the majority of cases, with 10 out of 16 best pipelines using one of these models as the classifier. Of those 10, the optimal choice of RF variant is split, with 4 optimal pipelines using RF and the remaining 6 using ExtraTrees. This indicates that the additional randomness introduced by ExtraTrees does not always result in improved classification accuracy. We can also observe that ExtraTrees generally achieve the best median classification accuracy, regardless of the employed feature extraction method whereas RF only achieves similar performance when using CSP for feature extraction.

The differences between individuals are further highlighted by the variability in optimal parameter settings of the model. For instance, looking at optimal CSP settings, the optimal number of components is highly variable, with some only using 3 components, while other pipelines require 9 components for optimal results. Generally, RF classifiers seem to work best with a high number of components, with the exception of the pipeline for participant 19, which only uses 3 CSP components.

For optimal RF and ExtraTrees settings, entropy is the most common optimal criterion for splitting decision tree nodes, with the exception of participants 15 and 19 using Gini Index, an alternative criterion which calculates the probability of a specific feature being classified incorrectly when its value is assigned randomly. The other RF parameters are more variable, with the maximum number of features used in a decision tree ranging from 0.25 (that is, using only 25% of features) to 0.75 and the optimal number of decision tree estimators ranging from 20 to 160.

Regarding the optimal filter frequencies, we can once again observe significant variability between individuals. Generally, filtering out low frequencies below 5 Hz seems to be essential to good accuracy, with the exception of (amputee) participants 01 and 03, whom benefited from the inclusion of lower frequencies. In both cases, the highest high-pass setting of 41 Hz was also the optimal choice. Filtering out frequencies below 8 Hz is the most common lower boundary, with eight optimal pipelines using this lower bound. Including higher frequencies appears to be beneficial in some cases, while decreasing performance in others. Eight optimal pipelines filter out frequencies above 32 Hz, while 5 use a higher bound of 41 Hz.

Although MI activity, which is commonly used to control BCI systems, is generally observed between 8 and 30 Hz (Pfurtscheller and Neuper, 2001), retaining higher and/or lower frequencies during preprocessing appears to increase decoding accuracy in some cases. In other cases, MRCP-related frequencies are more relevant for detecting motor intention than those for MI. Assessing whether a user's movement intention is most strongly present in MRCP frequencies or MI frequencies could therefore prove useful when determining the frequency ranges to use for a specific user. This means that user-specific frequency bands are necessary, as previously shown by Pfurtscheller and Neuper (2001), among others (Ang et al., 2008; Gaur et al., 2018; Nakagome et al., 2020).

The practical implication of this large variability means that it would be worthwhile to evaluate multiple possible pipelines in real-life situations. Next to selecting the optimal pipeline components and filtering boundaries, the parameters of these components should also be calibrated to the user. As long as there is no general model that can perform well on all users, it would be more practical to determine the best pipeline and settings for each user on an individual basis. While time-consuming, this process could be automated. This highlights the importance of developing methods for automated user-specific calibration to reduce the necessary calibration time and effort.

Since the data consisted of matched trials for both individuals with an amputation and able-bodied individuals, a comparison could also be made between the two groups. For this study, the analysis was limited to a comparison of the BCI performance between the two groups and the trends in the optimal pipelines. We found that there are no statistically significant differences in classification accuracy between the two groups. Similarly, no trend could be identified in terms of optimal pipeline composition. These results seem to indicate that the effect of neuroplasticity is limited when it comes to decoding movement intention from EEG in healthy limbs.

To further compare the differences in EEG activity for individuals with an amputation and able-bodied individuals, the GFP was visualized in a grand average over both groups. The resulting figures display the typical activity related to movement for both movements and similar waveforms for both groups (Figure 3).

One of the major limitations of this study pertains to the type of data that was collected. Since the participants only performed actual movements with their healthy limb, motor imagery in the amputated limbs is not represented. Furthermore, the BCI methods used in this study were limited to basic methods to show the feasibility of discriminating different single-joint lower limb movements from EEG, and did not investigate state-of-the-art methods. The evaluation of the methods was also limited to offline decoding accuracy for the best pipeline settings. Since the chosen time windows for the EEG input also include data from 2 s after movement onset, the models that were trained according to our method would not be suited for real-time decoding. This delay of 2 s would inhibit the user's sense of agency, removing the main advantage of using BCI. Furthermore, since the evaluation of the models was performed on a high-end desktop, the timing results would not be representative for the real-world setting. Another important aspect to note is that the code used was not optimized for fast execution as this was outside of the scope of this study. Therefore, extensive benchmarks of state-of-the-art methods are necessary to assess which specific methods would be the most suitable for real-time decoding.

Future research avenues include expanding the dataset with more data samples on the one hand and including imagined movement of the missing limb for individuals with an amputation on the other hand. This would allow for a more comprehensive comparison of the two populations, enabling more advanced analysis which takes into account neural activity related to actual movement and MI of the lower extremities. A better understanding and more data to train ML models would enable the development of more sophisticated BCI decoding pipelines. As we did not find significant differences in BCI decoding between the able-bodied group and the group of individuals with an amputation, it would be interesting to investigate whether it is possible to use a decoding pipeline that was trained with data from able-bodied individuals in a neuroprosthetic control system. If this is possible, the data collection to train the decoding models for a prosthetic control system would become much easier.

Furthermore, a deeper investigation of CSP and RF methods in the context of EEG could provide an improved understanding of neural activity in the lower limb, complementing classical neuroscientific methods, such as source imaging (Michel and Brunet, 2019). One of the main advantages of both CSP and RF is their explainability. CSP components can be interpreted to localize activity related to event related potentials (Congedo et al., 2016) and RF methods can be used for empirically ranking features based on their importance in classification (Breiman et al., 2017). Another possible analysis regarding the location of EEG activity could be investigating the effect of only using certain subsets of EEG channels in decoding. Together, these analyses could provide valuable insights into the neural mechanisms involved in lower limb movement.

Possible advanced methods that would be worth investigating should include transfer learning methods (Wan et al., 2021) to assess between-subjects transfer of BCI decoding capabilities, which could enable the implementation of a general purpose BCI classifier for lower limb movement. This will likely require the use of more advanced ML methods, such as deep learning (Roy et al., 2020) or automated fine-tuning of feature extraction methods (Zhang et al., 2017; Jao et al., 2018; Gurve et al., 2020). Deep learning seems to be an attractive prospect, as it has already been shown to be successful in lower limb decoding (Nakagome et al., 2020; Tortora et al., 2020b). Automated detection of optimal frequency bands for user-specific calibration could also contribute to further improving decoding performance and facilitating the deployment of BCI systems.

In real-world BCI settings, interactions are much more complex than single-joint movements, and additional constraints, such as decoding time, come into play. While it was shown in previous work that real-time decoding should not be an issue with these methods (Even on embedded hardware; Tam et al., 2020; Yu et al., 2022), the accuracy decreases with complexity and number of movements to decode. This is another reason, why state-of-the-art methods would likely be more appropriate. Combining EEG with other control signals, such as EMG, in a hybrid BCI system (Banville and Falk, 2016; Wöhrle et al., 2017; Li et al., 2019; Hooda et al., 2020; Tortora et al., 2020c), and further developing artifact removal methods (Gorjan et al., 2022), could prove necessary to achieve the balance in reliability and decoding speed that are required for real-world BCI control. Therefore, investigating real-time decoding with state-of-the-art models is essential to move toward practical BCI decoding in real-world applications. Assessing the real-time performance of these decoding methods will require more extensive evaluation methods than those used in the current article. For example, to evaluate model performance on data that was not involved in the optimal parameter selection process, a separate test set should be acquired.

Another crucial aspect that becomes an added concern with real-world BCI applications is the design of the control system. As we decode single-joint movements, these could be used to trigger discrete actions beyond the coordinated movements of regular gait cycles. For example, detection of ankle dorsiflexion could be used to press and release the clutch of a car with a prosthesis during driving. With real-world applications, user experience and ease-of-use become the most important requirements. Investigating advanced real-time decoding methods and developing a prototype real-time decoding pipeline is therefore an important final step toward bringing real-world BCI control of lower extremity neuroprostheses closer to reality.

## 5. Conclusion

The presented decoding results show that BCI control of a lower limb prosthesis should be feasible. We trained a selection of basic decoding methods and found that these methods can achieve acceptable decoding performance, which is on par with previous work on similar settings (Gu et al., 2021; Asanza et al., 2022). This leads us to believe that further improvements can be made by using more advanced methods. The development of BCI control of lower extremity prostheses would improve the sense of agency for the user and provide more natural control of the device.

In addition, when comparing the average decoding performance of the optimal pipeline for each participant with a statistical test, no significant differences between groups of people with amputations and healthy people could be found. This suggests that one could build a decoding pipeline for prosthetic device control using data from non-disabled individuals. However, more research is needed on various aspects to confirm these hypotheses and determine the most appropriate decoding methods.

## Data availability statement

The datasets presented in this article are not readily available due to highly private personal biometric data. Participants need to give additional consent to publicly share their data. Requests to access the datasets should be directed to kevin.de.pauw@vub.be.

## Ethics statement

The studies involving human participants were reviewed and approved by Medical Ethics Committee of the University Hospital of Vrije Universiteit Brussel (B.U.N. 1432020000041). The patients/participants provided their written informed consent to participate in this study.

## Author contributions

AD wrote most of the article and performed most of the analyses. EL performed the acquisition of the data for this study. EL and AM assisted with the analyses and contributed content to the text pertaining to their work. UM, FG, OR, RM, and KD provided feedback over multiple iterations and are principal investigators of the involved projects. All authors reviewed the manuscript and approved its submission.

## Conflict of interest

The authors declare that the research was conducted in the absence of any commercial or financial relationships that could be construed as a potential conflict of interest.

## Publisher's note

All claims expressed in this article are solely those of the authors and do not necessarily represent those of their affiliated organizations, or those of the publisher, the editors and the reviewers. Any product that may be evaluated in this article, or claim that may be made by its manufacturer, is not guaranteed or endorsed by the publisher.
